# Hexavalent vaccines in preterm infants: an update by Italian Society of Pediatric Allergy and Immunology jointly with the Italian Society of Neonatology

**DOI:** 10.1186/s13052-019-0742-7

**Published:** 2019-11-19

**Authors:** E. Chiappini, C. Petrolini, C. Caffarelli, M. Calvani, F. Cardinale, M. Duse, A. Licari, S. Manti, A. Martelli, D. Minasi, M. Miraglia Del Giudice, GB. Pajno, C. Pietrasanta, L. Pugni, MA. Tosca, F. Mosca, GL. Marseglia

**Affiliations:** 1 SODc Malattie Infettive AOU Meyer, Dipartimento di Scienze della Salute, Università di Firenze, Firenze, Italy; 2 Dipartimento di Scienze della Salute, Università di Firenze, Firenze, Italy; 3 Clinica Pediatrica, Dipartimento di Medicina e Chirurgia, Università di Parma, Parma, Italy; 4 Dipartimento di Pediatria, Ospedale S. Camillo-Forlanini, Roma, Italy; 5 UOC Pediatria, Servizio di Allergologia e Pneumologia Pediatrica, Azienda Ospedaliera-Universitaria “Consorziale-Policlinico”, Ospedale Pediatrico Giovanni XXIII, Bari, Italy; 6 Dipartimento di Pediatria, Policlinico Umberto I, Università Sapienza di Roma, Roma, Italy; 7 Clinica Pediatrica, Fondazione IRCCS Policlinico “S. Matteo”, Università di Pavia, Pavia, Italy; 8 Dipartimento di Medicina Clinica e Sperimentale, Unità di Broncopneumologia Pediatrica, Università di Catania, Catania, Italy; 9 UOC Pediatria, Azienda Ospedaliera G. Salvini, Ospedali di Garbagnate Milanese e Bollate, Milano, Italy; 10 Unità Pediatria, Ospedale di Polistena, Reggio Calabria, Italy; 11 Dipartimento della Donna, del Bambino e di Chirurgia Generale e Specialistica, Università della Campania Luigi Vanvitelli, Napoli, Italy; 12 Dipartimento di Pediatria, Unità di Allergologia, Università di Messina, Messina, Italy; 13 Terapia intensiva neonatale, Fondazione IRCCS “Ca’ Granda”, Ospedale Maggiore Policlinico; Dipartimento di Scienze Cliniche e di Comunità, Università di Milano, Milano, Italy; 14 Allergologia Pediatrica, Istituto Giannina Gaslini, Genova, Italy

**Keywords:** Vaccines, Preterm infants, Hexavalent vaccines

## Abstract

Hexavalent vaccines, protecting against six diseases (diphtheria, tetanus, pertussis [DTaP], poliovirus, hepatitis B virus [HBV], and *Haemophilus influenzae* type b [Hib], are routinely the standard of care in Europe. The use of combined vaccines allows the reduction of number of injections and side effects, the reduction of costs, and the increase in adherence of the family to the vaccination schedule both in terms of the number of doses and timing. The safety profile, efficacy and effectiveness of hexavalent vaccines have been extensively documented in infants and children born at term, and data are accumulating in preterm infants. Hexavalent vaccines are particularly important for preterm infants, who are at increased risk for severe forms of vaccine preventable diseases. However, immunization delay has been commonly reported in this age group. All the three hexavalent vaccines currently marketed in Italy can be used in preterm infants, and recent data confirm that hexavalent vaccines have a similar or lower incidence of adverse events in preterm compared to full-term infants; this is likely due to a weaker immune system response and reduced ability to induce an inflammatory response in preterm infants. Apnoea episodes are the adverse events that can occur in the most severe preterm infants and / or with history of respiratory distress. The risk of apnoea after vaccination seems to be related to a lower gestational age and a lower birth weight, supporting the hypothesis that it represents an unspecific response of the preterm infant to different procedures. High seroprotection rates have been reported in preterm infants vaccinated with hexavalent vaccine. However, a lower gestational age seems to be associated with lower antibody titres against some vaccine antigens (e.g. HBV, Hib, poliovirus serotype 1, and pertussis), regardless of the type of hexavalent vaccine used. Waiting for large effectiveness studies, hexavalent vaccines should be administered in preterm infants according to the same schedule recommended for infants born at term, considering their chronological age and providing an adequate monitoring for cardio-respiratory events in the 48–72 h after vaccination, especially for infants at risk of recurrence of apnoea.

## Introduction

**Fig. 1:**
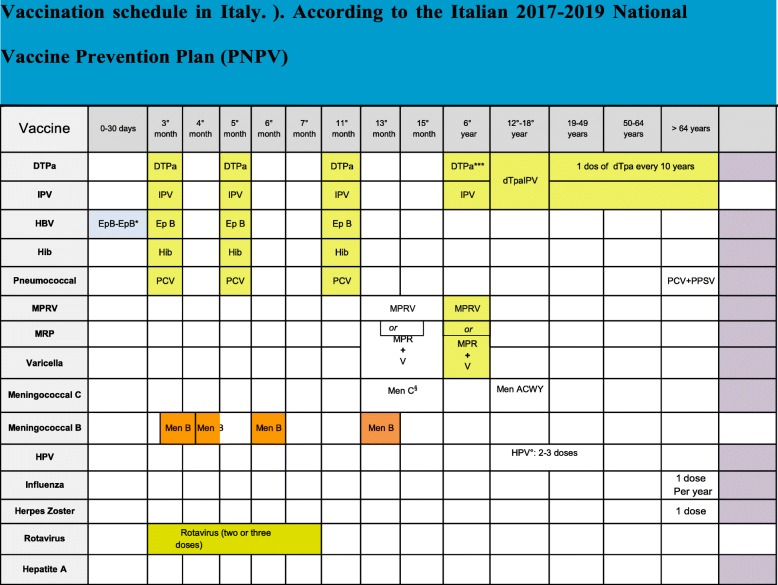
Vaccination schedule in Italy.). According to the Italian 2017–2019 National Vaccine Prevention Plan (PNPV)

**Fig. 2:**
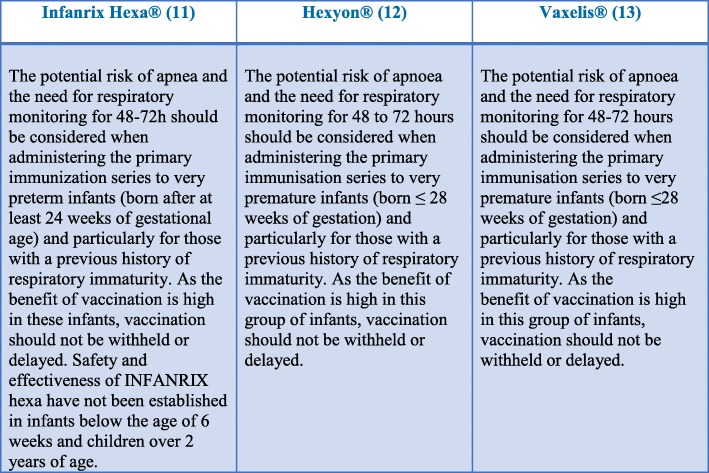
Infanrix Hexa®, Hexyon® e Vaxelis®. Summary of product characteristics as reported by EMA (European Medicine Agency). Paragraph concerning safety in preterm infants, including those born with ≤28 weeks of gestational age and/or recent history of respiratory distress

In Italy, as well as in most European countries, hexavalent vaccines, protecting against six diseases (diphtheria, tetanus, pertussis [DTaP], poliovirus, hepatitis B virus [HBV], and *Haemophilus influenzae* type b [Hib]), are routinely the standard of care [1–6]. Combined vaccines allow the reduction of the number of injections and number of side effects, the reduction of costs and the increase in adherence of the family to the vaccination schedule both in terms of the number of doses and timing [7, 8]. As a consequence, in countries which have adopted a program based on hexavalent vaccines, the coverage rates in the first year of life are high (from 90 to 99%) and higher than those obtained in countries that use other multivalent vaccines as the only option or as an alternative to hexavalent vaccination (70–90%) [8]. According to the Italian 2017–2019 National Vaccine Prevention Plan (PNPV) schedule (Fig. 1), the primary immunization cycle with hexavalent vaccines includes 3 doses, to be administered at 3–5-11 months of age, followed by booster doses in preschool age (at 6 years) with DTPa and inactivated poliovirus vaccines (DTPa-IPV) and with dTaP-IPV in adolescents (12–18 years) and adults, to be repeated every 10 years [9]. This strategy has been associated with a dramatic reduction in the number of cases of infectious diseases targeted by the hexavalent vaccines in Italy [10]. Currently, three hexavalent vaccines are marketed in Italy, Infanrix Hexa®, Hexyon® and Vaxelis®, which can be administered in preterm infants. The European Medicines Agency (EMA) authorizes the use of the three hexavalent vaccines even in the most severe preterm infants ( [11–13] Fig. 2).

In term infants the three hexavalent vaccines have been proved to be not inferior to each other for immunogenicity and safety profile, even in co-administration with anti-pneumococcal and anti-rotavirus vaccination [11–15]. Moreover, large effectiveness studies have been performed in several European countries (including Sweden, Denmark, and Germany) concerning diseases such as pertussis and invasive Hib [16–18]. The safety data collected in the clinical studies on hexavalent vaccines have also demonstrated good tolerability of these vaccines, confirmed by phase IV post-marketing surveillance studies, reporting a higher percentage, but not statistically significant, of fever and mild, transitory, local symptoms compared to vaccines with fewer components [11–13, 19, 20]. It was estimated that in 2018 approximately 500,000 preterm infants have been vaccinated with Hexyon®, while a similar number of preterm infants in the same year were vaccinated with Infanrix Hexa® and Vaxelis® [8]. A recent publication by a panel of Italian experts from the Italian Society of Hygiene and Preventive Medicine, The Italian Society of Pediatrics and the Italian Federation of Pediatrics confirmed that all the three hexavalent vaccines can be used in preterm infants (Tables 1 and 2), [7]. The panel underlined that no delay in undertaking the national vaccination schedule can be justified in preterm infants, as well as reluctance in the co-administration of other vaccines, including anti-pneumococcal vaccine and anti-rotavirus vaccines. Once the absolute contraindications are excluded, vaccination should be executed basing on the chronological age of the child [6, 21–24]. This is particularly important considering that high prevalence of sepsis [25–28], pertussis [29], and other diseases [30, 31] have been reported in preterm infants.

**Table 1:** Sub-categories of preterm birth, based on gestational age. From WHO. Preterm birth. Fact sheet. 2016. www.who.int/mediacentre/factsheets/fs363/en/. (modified)

Subcategory of preterm birth	Gestational age
moderate to late preterm	32 to 37 weeks
very preterm	28 to 32 weeks
extremely preterm	< 28 weeks

Preterm is defined as babies born alive before 37 weeks of pregnancy are completed

**Table 2:** Comparison of indications and use of the three hexavalent vaccines (from Orsi et al., 2018 [7], modified)

	Infanrix Hexa® [11]	Hexyon® [12]	Vaxelis® [13]
Hib PRP	10 μ g conjugated to tetanus toxoid	12 μ g conjugated to tetanus toxoid	3 μ g conjugated to membrane protein meningococcus (OMP)
Pertussis PT	PT 25 μ g FHA 25 μ g PRN 8 μ g	PT 25 μg FHA 25 μg	Pertussis PT 25 μ g FHA 25 μ g PRN 8 μ g PT 25 μ g FHA 25 μ g PT 20 μ g FHA 20 μg PRN3 μg FIM type 2.3: 5 μg
Diphtheric toxoid	Not less than 30 IU * average value	Not less than 20 IU * lower limit 95% CI	Not less than 20 IU * lower limit 95% CI
Tetanus toxoid	Not less than 40 IU	Not less than 40 IU	Not less than 40 IU
IPV polio	Inactivated virus Types 1, 2, 3	Inactivated virus Types 1, 2, 3	Inactivated virus Types 1, 2, 3
Hepatitis B HBsAg produced in	*Saccharomyces cerevisiae*	*Hansenula polymorpha*	*Saccharomyces cerevisiae*
Ready to use No Yes Yes	No	Yes	Yes
Co-administration with other vaccines included in the national schedule	Yes	Yes	Yes
Minimum age	Yes	Yes	Yes
Minimum age	Not specified	6 weeks	6 weeks
Antibody persistence studies	Yes	Yes	Yes
Effectiveness data	Yes	Yes	Not available

Despite the national and international recommendations, however, several studies have shown low vaccination rates and delays in the majority preterm infants [32–35]. A post-marketing surveillance study on vaccination with Infanrix Hexa® and Hexyon® vaccines found that only 57.6% of preterm infants of the Puglia in 2017 had been vaccinated by their 90th day of life [36]. The ACTION follow-up project (Access to Intensive Care and Neonatal Obstetrics, Access to Obstetrical and Neonatological Intensive Care follow-up project) evaluated preterm infants at 22–31 weeks of EG in 5 Italian regions in 2003–2005 (Friuli-Venezia-Giulia, Tuscany, Marche, Lazio, Calabria) and showed that the delay in starting vaccination was correlated to a lower weight and EG at birth, to a second hospitalization after discharge from the neonatal intensive care unit (NICU), to maternal / paternal unemployment, the number of children within the family, lower socio-economic status, positive anamnesis for cerebral palsy and ethnicity other than Caucasian [33]. Some possible reasons of the vaccination delay in preterm infants concern safety and efficacy of hexavalent vaccines in these children [37–40]. Hereby, we reviewed and summarized the recent literature regarding safety, efficacy and effectiveness of hexavalent vaccines in preterm infants, which further support the national and international recommendations.

## Methods

A systematic search of the literature published from 1 January 2008 to 30 June 2019 was performed on PubMed MEDLINE and Cochrane Library databases, using the following Boolean expression (“infant, preterm” [MeSH Terms]) AND “vaccination” [MeSH terms] AND “hevavalent vaccines” [MeSH terms]) and selecting only articles published in English. The reference of articles retrieved by this search strategy were also examined in order to recover any further relevant publications. A first screening of the selected articles was carried out on the basis of the title and the abstract. We included all prospective or retrospective observational studies and clinical trials regarding efficacy, effectiveness or safety profile of hexavalent vaccines in preterm infants. Studies that carried out a comparison between the hexavalent vaccination in full-term and preterm infants and studies in which hexavalent vaccines was co-administered with other vaccination types (e.g. anti-pneumococcal, anti-rotavirus, anti-VRS, anti-influenza) were included. The non-pertinent articles, commentaries, letters, case series concerning < 10 children cases, reviews, duplicates and articles not written in English were excluded. For each study the following data were evaluated and summarized in the tables: year of publication, study design, gestationale age (GA) of the population, number of study included, type of vaccination administered, outcomes (e.g. laboratory parameters for immunogenicity, clinical indicators for efficacy and reported adverse events for safety), follow-up period and any study bias / limits.

## Results

Initially, 101 articles were retrieved by the search strategy. Sixteen articles regarding hexavalent vaccination in preterm infants have been identified, of which 13 and 5 concerned safety ([39, 40, 42]; Table 3) and efficacy/effectiveness (Table 4), respectively. In all these studies hexavalent vaccines were administered according to the primary 3-dose immunization schedule, as recommended by the Centers for Disease Control and Prevention [43]. Among the 13 safety studies, 11 had hexavalent vaccination [39, 40, 42], as main objective, while 2 studies had, as their main objective, other types of vaccines (anti-pneumococcal and anti-rotavirus) in co-administration with hexavalent vaccines. In 9 out of 11 safety studies on hexavalent vaccines, other vaccines were co-administered [39, 40, 42] in the same vaccination session. Regarding the safety of hexavalent vaccines in preterm infants, several studies showed a similar or lower incidence of both local and systemic adverse events in preterm compared to full-term infants, probably due to the lower ability to induce an inflammatory response in preterm infants, even when co-administered with pneumococcal vaccine None of the serious adverse events observed in these studies were considered causally related to the vaccination. Apnoea and alterations in reactivity in preterm infants were the most frequently reported adverse events. Numerous studies have shown an increase in the incidence of apnoea episodes in preterm infants after hexavalent vaccination [40–42]. Preterm infants who have experienced apneoa episodes after hexavalent vaccination are generally those in more critical clinical conditions (e.g. previous late-onset sepsis), who required greater support through continous positive airway pressure, and who have a positive history for similar episodes, particularly in the 24 h prior to the administration of the vaccination [40–42]. A lower birth weight (< 2 kg), a lower GA (≤ 31 weeks) and chronological age (< 67 days), a positive history for similar episodes and hospitalization for complications related to prematurity are risk factors of recurrence of post-vaccination apnoea [40] also at the second dose of the vaccination schedule with variable percentage in the datasets (4.4–18%) [39, 40]. However, a causal relationship between the vaccine and the appearance of cardio-respiratory events continues to be, widely debated. The available studies are often retrospective, with no control group and with numbers of included children too limited to demonstrate a statistically significant difference in the incidence of these events in preterm infants. Furthermore, it is difficult to distinguish apnoea related to vaccination from those due to the clinical instability of preterm infants due to other associated comorbidities (e.g. periventricular hemorrhage, bronchopulmonary dysplasia or late onset sepsis). Indeed, the only available prospective randomized controlled trial by Carbone et al. suggests that the hexavalent vaccine administration is not associated with cardio-respiratory events, showing no difference in the frequency and severity of apnoea / bradycardia episodes in those who had received vaccination compared to controls. Given the potential risk of apnoea reported in several studies, however, preterm infants still hospitalized at 2 months of life should be vaccinated before discharge from the NICU with a clinical and respiratory monitoring for the 48–72 h following vaccination, particularly with regard to the very low birth weight (VLBW) preterm with a positive medical history for cardio-respiratory events, especially in the 24 h prior to the administration of the vaccine. If a cardio-respiratory episode has occurred after the first vaccine dose, the second dose should be administered in a protected environment in those individuals at high risk of recurrence.

**Table 3:** Studies concerning the safety of hexavalent vaccine co-administered with other types vaccine in pre-term infants

Author (year)	Study design	Number of children	Gestational age (EG) in weeks (s)	seat	Vaccine	Target	Follow-up (FU) after the vaccine	Results	Bias
Wilińska et al. (2016)	Observational perspective	138	73 ≤ 28 s 65 > 28 s	Poland	DTPa, IPV, HBV, Hib, Co-administration: PCV7	Evaluate incidence of adverse events after vaccination by monitoring CR parameters and body temperature	72 h	• Apnea and reactivity alterations are the most frequent adverse events (4 and 9% respectively) • Those born preterm who present apnea ano have experienced in a statistically more mind frequently lateonset sepsis (*p* = 0.028) and a more prolonged use of *continuous positive air pressure* (CPAP) (*p* = 0.033)	• No group of control • Limited sample
DeMeo et al. (2015) [42]	Multi-center cohort retrospective	13.926	≤ 28 s	United States of America	DTPa, IPV, HBV, Hib Co-administration: PCV7	Evaluate the number of tests for sepsis (blood culture sampling), the increased need for respiratory support, convulsions and death in 3 days after the vaccination	3 days	• The incidence of findings for sepsis and the need for respiratory support increases after vaccination • Children with an EG of 23–24 weeks demonstrate an increased incidence of sepsis tests and an increased need for respiratory support compared to children with major EG (2728 weeks) • Gram-positive + A history of sepsis is associated with a hearing ished fre ence of investigations for sepsis after vaccine administration	• *Healthy vaccinated* effect • Clinicians more readily document adverse events that occur in the immediate vicinity of the vaccine administration
McCrosan et al. (2015)	retrospective tivo	344	< 37 s	Ireland	DTPa, IPV, HBV, Hib, Co-administration: PCV7	Evaluate the safety of preterm vaccines		No child presented adverse events	• Studio retrospotting scope • Not clear the period of follow-up
Anderson et al. (2012)	retrospective tivo	203	≤ 28 s	Auslia	DTPa, IPV, HBV, Hib, Co-administration: PCV7	Evaluate apnea in the 48 h following vaccination to 2 months of life	48 h	• 17 preterm have presented a framework clinically compatible with apnea (incidence 8.4%) after vaccinations nation than 2 months • Children who have experienced apnea at 2 months of age have a statistically significant lower EG and a lower birth weight • No reaction to subsequent vaccination doses	• Limited sample • Lack of cardio-saturimetric monitoring in 50% of cases at the 4 month vacine dose
Clifford V et al. (2011) [39]	Retrospect vo observational	46	38 < 37 s 8 ≥ 37 s	Auslia	DTPa, IPV, HBV, HiB, Co-administration: PCV7, rotavirus	• Evaluate the occurrence of adverse events in the 48 h following the vaccine at 2 and 4 months of life • Investigate any risk factors for apnea recurrence	48 h	• 35/38 preterm has apnea after the 2 month vaccine, 3/38 after the 4-month vaccine • 7/38 (18%) has a recurrence of apnea • A lower birth weight (*p* = 0.04) and hospitalization due to complications related to prematurity (*p* = 0.01) increased no risk of recurrence of apnea • No child with recurrent apnea post-vaccination at four months has presented an apnea after the third vaccine dose than 6 months	• Limited sample • Studio retrospotting scope
Furck et al. (2010)	Observational perspective	473	< 37 s	Germany	DTPa, Hib, HBV, IPV Co-administration: PCV7	Evaluate the adverse events within the next 48 h the vaccine	48 h	• The frequency of adverse events is 10.8 and 2.8% for apnea / bradycardia and local / fever reactions, respectively • Incidence of apnea / bradycardia increases in co-administration with PCV7 but not in a statistically significant manner • The risk of experiencing episodes of apnea decreases with increasing EG • Fever is statistically more significant in children with grade 3–4 cerebral haemorrhage or with leucomalacia periven tricular (OR 8.7 and 8.2 respectively)	• The 3 groups do not have the same number of children • The EG at the time of vaccination is reduced with advancing years
Hacking et al. (2010)	retrospective cohort study	411	27 s	Auslia	DTPa, Hib, HBV, IPV Co-administration: PCV7, rotavirus	Assess the need for support respiratory (CPAP) or of ventilation in positive international pressure sender (IPPV) within 7 days after the vaccine at 2 months	7 days	• 22/411 (5%) pre-term experience a worsening of respiration in the following 3 days the vaccine attributable only to immunization • Children who needed respiratory support after the disease have a greater previous incidence of sepsis (*p* = 0.02) and a greater average cumulative time of use of CPAP before the vaccine (*p* = 0.03)	
Klein et al. (2010)	Prospective *self-controlled case series approach*	83	33 < 37 s 50 ≥ 37 s	United States of America	DTPa, Hib, HBV, IPV, Co-administration: PCV7	Describe the AE 30 days after each vaccination dose confrontandone frequency in pretermiit and born at term	30 days	• No adverse events reported in the 2 groups • The *self-controlled case series* analysis showed no increase in adverse events in full-term and preterm births after no vaccination	Limited sample
Carbone et al. (2008)	Perspective, randomized, controlled, double-blind, multicentric	197	< 37 s	United States of America	DTPa, IPV, Hib, HBV	Evaluate the increase in CR events after vaccination in preterm	48 h	No increase in CR events in the vaccine group compared to the control group	No long-term follow-up in the control group
FlatzJequier et al. (2008) [40]	retrospective tivo	135	< 32 s	Switzerland	DTPa, IPV, HiB, HBV, Co-administration: PCV7, anti VRS	Evaluate the frequency of CR events later hexavalent vaccination in VLBW children in the next 48 h the second dose of vaccine	48 h	• 34/135 VLBW (25%) presented a CR event after a 2-month vaccination dose • 6/33 who had had a reaction after the 1 ^-day^ dose needed a medical intervention (eg oxygen supplementation, tactile stimulation, mask ventilation) after the vaccine at 4 months • No children showed a CR event after the third vaccine dose. • A similar p recourse CR post vaccinations final event is the factor risk of recurrence	Retro-view study
Klein et al. (2008)	retrospective tivo	497	456 ≤ 30 s 41 31–41 s	United States of America	DTPa, IPV, HBV, Hib Co-administration: PCV, in fluence	Evaluate the factors associated with post-vaccination apnea	48 h	• 95% post-vascular apnea (62/65) occurs in preterm born ≤31 weeks of EG • The bivariate analysis shows that the presence of pre-vaccination apnea is markedly associated with the appearance of post-vaccinal apnea (*p* < 0.0001) • Multivariate analysis found that a SNAP-II > 10 (AOR: 4.2; 95% CI: 1.2–14.3), the chronological age < 67 days (AOR: 2.3; 95% IC: 1.1–4.8) and weight < 2 kg (AOR: 2.1; 95% CI: 1–4.5) They are associated with the apneas post-vaccinal	
Omenaca et al. (2012)	Prospective, multicentric, randomized, controlled, double-blind trial	250	≥ 27 < 37	France, Portugal, Poland, Spain	Rotavirus Co-administration: DTPa, IPV, Hib, HBV	Evaluate the incidence of adverse events at 15 and 31 days after vaccination and any serious adverse events	31 days	• Similar frequency of adverse events reported in the vaccine group and in the placebo group (*p* = 0.266) • In the 31 days following the vaccination dose is STAto reported at least one adverse event in both the vaccinated for rotavirus group than in the group placebo • The percentage of all adverse events including those of grade 3 reported 15 days after the vaccine is similar in both groups (*p* > 0.05) with irradiation as the most common event	
Omeñaca et al. (2011)	Prospective trial	286	50 27–30 s 87 31–36 s 149 ≥ 37 s	Spain, Greece	PHID-CV Co-administration with DTPa, IPV, Hib, HBV	Evaluate the safety of PHiD-CV e of vaccines co-administered with 2–4-6 months and 16–18 months Evaluate the local and systemic adverse events 31 days after the vaccine dose and serious adverse events within 6 months following the booster dose	31 days (6 months for severe adverse events)	• The most frequently observed systemic adverse events are irritability, drowsiness, fever and loss of appetite, but the incidence of high-grade systemic adverse events is low (eg 0.8–1.5% with regard to fever> 39 °C in the 4 days following the doses of the primary cycle, 7.1% as regards the subsequent one the booster dose) • The incidence of grade 3 local adverse events is low (< 5.3%) in both groups but greater after the booster dose in full-term births • No severe adverse events were reported to be correlated with the vaccine • It was noted an episode of apnea in preterm infants after the first dose vacSino but has not been considered to be related to the vaccine and left no sequelae	

*DTPa* Diphtheria, tetanus, acellular pertussis, *IPV* Polio inactivated vaccine, *HBV* Hepatitis B vaccine, *PCV7* Heptavalent pneumococcal vaccine, *PCV13* Pneumatic anti-pneumococcal vaccine, *Hib H. influenzae* type b vaccine, *SNAP-II* Score for Neonatal Acute Physiology II, *VLBW* Very Low Birth Weight, *PHID-CV* Decavalent pneumococcal vaccine (PCV10) conjugated to the non-typable D protein of *H. influenzae*, *CR* Cardio-respiratory, *d* days

**Table 4:** Studies concerning the immunogenicity of hexavalent vaccination in preterm

Author (year)	Study	Number of children	Gestational age (EG) in weeks (s)	Sed	Vaccine	Target	Results	Bias
Vermeulen et al. (2013)	Prospective cohort observation	68 • 22 immunized with vaccine cellulare (Pw) • 24 immunized with 2-component acelular vaccine (Pa-2) • 22 immunized with 3-component acelular vaccine (Pa-3)	< 31 s	Belgium	3 types: Pw, Pa-2, Pa-3	Evaluate the 1-year specific cellular response in the preterm by cytokine secretion after antigenic stimulation	• More than half of the preterm vaccinated with Pw or Pa-2 develops a response at 3 and 6 months • IFNɤ to FHA and PT • No effect of the booster dose on FHA or PTinduced IFNzione secretion in the 3 groups • The Pa vaccine induces a greater secretion of Th2 cytokines in response to FHA and PT, compared to children vaccinated with Pw	Limited sample
Omeñaca et al. (2011)	Prospective	286	• Group I: 27–30 s • Group II: 31–36 s • Group III: ≥ 37 s	Spain, Greece	PHiD-CV Co-administration DTPa, IPV, HBV, Hib, PCV	Evaluate the immunogenicity of PHiD-CV at 2, 4, 6 months by evaluating the antibody titre as OPA or GMC 1 month after the primary vaccy cycle and 1 month after the booster dose	One month after the primary vaccination cycle and the booster dose, all bambinii serum were protected against the antigens of vaccini coadministered	
Omeñaca et al. (2011)	Phase IIIb perspective, controlled, multicentric	309	• 56 group I: ≤ 31 s • 107 group II: 32–36 s • 150 group III: ≥ 37 s	Spain	Hib-MenC-TT to 2, 4, 6 months and 16–18 months Co-administration DTPa, IPV, HBV, Hib, PCV, rotavirus	Evaluate the immunogenicityof Hib-MenC-TT in preterm by measuring the specific antibody titer 1 month after the third dose and 1 month after the booster dose	• The percentage of subjects with a concentration of anti-PRP antibodies compatible with seroprotection is ≥99% in all groups • The booster dose induces a marked increase in anti-PRP GCM, after a reduction in the percentage of subjects with seroprotective titres before the booster dose • At least 97.5% of the subjects in each group have concentrations of anti-H Bs antibodies > 10 mIU / mL at 1 month after the third vaccination dose • The titer of anti-HBV antibodies after dose 3 is significantly lower in preterm than group I compared to those born with larger EGs	
Klein et al. (2010)	Observational perspective	88 33 ≤ 33 s 50 ≥ 37 s	≤ 31.3–39.5 s	United States of America	DTPa, IPV, HBV, Hib PCV co-administration	Compare the humoral and cellular response of preterm vs full term babies after the primary vaccination cycle	• Preterms and those born at term develop comparable levels of memory response of T cells to type 3 polioviruses • With regard to lympho-monocellular proliferation Preterms present less frequently a positive stimulation index compared to those born at term (p = 0.03) • All subjects have serumprotective antibody titers for the 3 types of poliovirus • The GMC towards the sierotipo 1 polio was significantly lower in pretermiit compared to those born to ter mines	
Omeñaca et al. (2010)	Prospective trial	182	93 < 37 s 89 ≥ 37 s	Spain	DTPa, IPV, HBV, Hib	Evaluate the response to hepatitis B vaccine in preterm after the primary vaccination cycle and the booster dose	• 93.4 and 95.2% of preterm and full-term babies respectively show seroprotection against HBV after the primary vaccination cycle • The GMCs for HBV after primary cycle are lower in the Group of preterm born than in the group of term births, although not in a statistically significant manner • 6 preterm (6.59%) respond neither to the primary cycle nor to the booster dose • Non-responders have an EG ≤ 31 s of which 2 are severe IUGR	

*DTaP* Diphtheria, tetanus, acellular pertussis, *IPV* Polio inactivated vaccine, *HBV* Hepatitis B vaccine, *PCV7* Heptavalent pneumococcal vaccine, *PCV13* Pneumatic anti-pneumococcal vaccine, *Hib H. influenzae* type b vaccine, *Hib-MenC-TT* Vaccine for *H. influenzae* type B-Neisseria meningitidis serogroup of type C, *PHID-CV* Decavalent pneumococcal vaccine (PCV10) conjugated to the protein

*D diH* Non-typable influenzae, *SNAP-II* Score for Neonatal Acute Physiology II, *PT* Pertussis toxin, *FHA* Phytohemagglutinin, *OPA* Opsonophagocytic activity, *GMCs* Geometric mean concentration, *PRP* Antipoliribosilribitolfosate

Focusing on the immunogenicity of hexavalent vaccines, high seroprotection rates have been reported in preterm infants. However, a lower GA seems to be associated with lower antibody titres against some vaccine antigens (eg HBV, Hib, poliovirus serotype 1, and pertussis), regardless of the type of hexavalent vaccine used. These data are in agreement with previous studies in which the seroprotection rates reached 98.7–100% in preterm infants [43, 44]. Lower seroprotection rates, although still high, have been reported with respect to pertussis and Hib (92.4 and 92.5% respectively) [44]. Although lower GA and birth weight seem to be associated with lower antibody titres against Hib, a booster dose of hexavalent vaccine was able to induce a protective serological response (evaluated as anti-PRP [anti-polyribosyl ribitol phosphate] antibody concentration > 1 μg/mL) in 98.2% of preterm infants, suggesting an adequate immunological memory in this population [45]. On the other hand, the ability to induce an adequate humoral response to HBV in preterm infants is still debated. In large studies the seroprotection rates for HBV were similar in very low birth weight (VLBW) and low birth weight (LBW) (93.7% vs. 94.9% respectively), However, seroprotection rates were reinforced (> 98%) by booster vaccination for all antigens except for HBs in VLBW children: only 88.7% of those had anti-HBs antibody concentrations > or = 10 mIU/mL, compared with 96.5% of LBW children (the difference was not statistically significant) [43]. In contrast, Omenaca et al. did not observe no difference in the humoral response to HBV vaccination in preterm infants of lower GA and birth weight neither after primary cycle nor after a booster dose [46]. Only one study investigated the T cell-mediated response in preterm infants, showing comparable levels of memory T lymphocyte response for poliovirus antigens in full-term and preterm infants after hexavalent vaccination, but the latter demonstrate several nonspecific and poliovirus-specific functional T cell limitations. Moreover, although all infants developed seroprotective poliovirus antibody titers, serotype 1 titers were lower among preterm infants (*P* = 0.03).

The immunogenicity of the hexavalent vaccines in co-administration with other types of vaccines was evaluated in several studies [47]. Effectiveness studies regarding the use of hexavalent in preterm infants are very scarce at the moment. Furthermore, in the available studies, a 3 + 1 schedule was used characterized, as recommended by the Centers for Disease Control and Prevention [48], while in some European countries, including Italy, a reduced (2 + 1) vaccination schedule is employed. Further studies are needed in order to define in greater detail the immunogenicity and effectiveness of hexavalent vaccines with reduced schedule in preterm infants, focusing also on the potential differences in the subgroup of severe preterm infants (< 29 weeks of EG), in order to assess the need for additional vaccine doses or for early administration of booster doses.

## Conclusions

Hexavalent vaccines administered to preterm infants display a good safety and immunogenicity profile even when co-administrated with other vaccines included in the Italian schedule. Summarizing the results of the present review, it should be bared in mind that:. the three hexavalent vaccines available in Italy have the same indications and can be administered in preterm infants; • although further studies are needed regarding their effectiveness, no delay in vaccination of clinically stable preterm infants is justifiable; • preterm infants must be immunized according to their chronological age, in the same time and manner as those born at term; • severe preterm infants who are still hospitalized at the time when they should be vaccinated should receive the first dose during the hospitalization, particularly in the case of preterm infants ≤31 weeks, with a birth weight < 2.0 kg, with previous episodes of apnoea / bradycardia before vaccination and / or with severe bronchopulmonary dysplasia, in order to provide monitoring for cardiorespiratory events for the 48–72 h following vaccination; • if a cardio-respiratory episode has occurred after the first vaccination dose, the second dose should be administered in a protected environment; • the specific recommendation for the administration of hexavalent vaccines in the most severe preterm infants (≤ 28 weeks) or with a recent history of respiratory distress, as reported by EMA, includes an additional respiratory monitoring for 48 to 72 h after the vaccination.

## Data Availability

Please contact author for data requests.
